# Evaluating the Role of School-Based Physical Activity in Mitigating Cardiometabolic Risk Factors in Children and Adolescents with Overweight or Obesity: A Systematic Review and Meta-Analysis

**DOI:** 10.3390/children12040439

**Published:** 2025-03-29

**Authors:** Dingmeng Mao, Bowen Li

**Affiliations:** Department of Sports Science, College of Education, Zhejiang University, Hangzhou 310058, China; mao_dm@zju.edu.cn

**Keywords:** cardiometabolic health, exercise, pediatric obesity, school, teenager

## Abstract

Background: Overweight or obese children and adolescents have a higher risk of developing cardiometabolic health problems compared with their healthy-weight peers, which are likely to progress to cardiovascular disease and are associated with a range of negative impacts. Objectives: To evaluate the effects of school-based physical activity (PA) interventions on cardiometabolic risk factors in children and adolescents with overweight or obesity. Method: A search of online databases was conducted to identify relevant studies up to 31 January 2025. Results: Eleven studies were included, involving 963 participants aged 7 to 18 years. School-based PA interventions have a significant effect size (ES) in reducing body fat percentage (ES = −0.43, *p* < 0.01), diastolic blood pressure (ES = −0.27, *p* < 0.05), triglycerides (ES = −0.38, *p* < 0.01), fasting blood glucose (ES = −0.60, *p* < 0.01), blood insulin (ES = −0.62, *p* < 0.01), and homeostatic model assessment-insulin resistance (ES = −0.58, *p* < 0.01) in overweight or obese students. However, no significant improvements were observed in body mass index, body mass index z-score, waist circumference, waist-to-height ratio, maximal oxygen consumption, systolic blood pressure, high-density lipoprotein cholesterol, low-density lipoprotein cholesterol, and total cholesterol. Conclusion: School-based PA interventions lasting 6 weeks longer, twice a week or more, can significantly mitigate some cardiometabolic risks of overweight or obese children and adolescents. Effective, targeted PA programs should be considered in the school setting to promote the cardiometabolic health of this vulnerable group.

## 1. Introduction

The prevalence of overweight and obesity in children and adolescents has become a major public health concern in the past decades [1]. The Lancet reported that global obesity has affected an alarming one billion individuals across the categories of children, adolescents, and adults, with the obesity rate among young adults being three times higher in 2022 compared to 1990 [2]. Obesity is a risk factor for several chronic diseases, including cancers [3] and type 2 diabetes [4]. Childhood obesity has a high potential to persist into adulthood [5] and can lead to reduced social participation, increased mental health problems, and a diminished overall quality of life [6]. Of note, overweight or obesity in children and adolescents is associated with multiple adverse metabolic health factors, such as insulin resistance, dyslipidemia, and hypertension, which can lead to an increased risk of cardiovascular disease [7]. Specifically, excess visceral fat contributes to metabolic abnormalities that result in insulin resistance, cardiometabolic disorders, and a raised risk of hypertension [8]. Moreover, central obesity (abdominal fat accumulation) is highly correlated with elevated levels of lipoprotein concentration and C-reactive protein, as well as with the development of atherosclerosis [9]. Thus, there is a need for early intervention in overweight or obese children and adolescents to promote their cardiovascular health.

Although medication can be part of obesity management, it is not a long-term solution and carries potential side effects [10]. Instead, physical activity (PA) is a well-established intervention for enhancing overall health and mitigating the risk of chronic diseases. Prior research has demonstrated that PA significantly contributes to improving lean tissue mass quality, boosting metabolic rate, strengthening cardiac muscle, and augmenting arterial system elasticity [11]. Additionally, several studies have identified a correlation between PA and cardiometabolic outcomes in children and adolescents [12]. For instance, recent evidence indicates that exercise interventions in overweight children yield improvements in cardiometabolic health by reducing adipose tissue, blood glucose levels, and waist measurement [13]. Yet, the optimal programs for promoting cardiometabolic health in overweight and obese children and adolescents remain to be elucidated.

According to Bronfenbrenner’s ecological systems theory [14], individual development is shaped by a multitude of interconnected environmental systems, with the school environment, as a pivotal microsystem, playing a crucial role. Research has highlighted that the school setting constitutes one of the most pertinent contexts for implementing interventions aimed at promoting healthy behaviors in younger populations, including PA [15,16]. Considering that school-age youths dedicate a substantial portion of their weekly time to school activities, this environment provides ample opportunities for engaging in exercise activities and cultivating regular PA habits. Numerous previous studies have rigorously assessed the efficacy of exercise interventions targeting overweight or obese children and adolescents, yielding promising outcomes [7,17]. However, ensuring the sustained engagement of students in PA interventions poses a challenge, particularly due to the difficulty in balancing academic obligations with the incorporation of these activities into their daily routines, with lots of studies being conducted in out-of-school settings, such as specialized training centers, medical clinics, or during vacation periods. Consequently, it is necessary to explore how to embed PA interventions specifically tailored for overweight and obese students within the framework of school routines.

Previous studies have evaluated the effectiveness of exercise in obesity prevention or metabolic risk among children and adolescents [18,19,20]. Still, the effectiveness of interventions on cardiometabolic risk factors specifically targeting overweight or obese students within the school setting is uncertain. Moreover, as the growing body of research on school-based PA interventions for overweight or obese children and adolescents, the findings are inconsistent and sometimes contradictory. A meta-analysis study showed that there was no statistically significant effect of school-based PA interventions on body mass index (BMI) and blood pressure (both systolic and diastolic) [21], while several recent studies have found some significant positive effects on a subset of cardiometabolic risk factors [22,23,24]. Additionally, the number and type of indicators of risk factors reported by established studies show some variation, which limits a comprehensive assessment of the effects of interventions on cardiovascular health. In recent years, several empirical research evaluating the effectiveness of school-based PA interventions among overweight and obese students have been published. Therefore, the present study aims to carry out a systematic review with meta-analysis to identify the effects of PA interventions in the school environment on cardiovascular risk factors in overweight or obese children and adolescents.

## 2. Materials and Methods

### 2.1. Protocol and Registration

This systematic review and meta-analysis followed PRISMA guidelines [25]. The protocol was registered with PROSPERO (number CRD42025645296).

### 2.2. Search Strategy

Three electronic databases (i.e., Web of Science, PubMed, and Embase) were systematically searched to identify relevant studies published between inception and 31 January 2025. The search items were grouped into five components: (1) PA (physical activity OR exercise OR workout OR exertion OR training OR movement OR sport), (2) cardiometabolic risk factor (cardiometabolic risk factors OR blood pressure OR waist circumference OR triglyceride OR lipids OR cholesterol OR glycemia OR glucose OR insulin OR metabolic syndrome), (3) age group of interest (child* OR adolescent* OR youth OR teenager OR student), (4) symptoms of overweight or obesity (overweight OR obesity*), and (5) surroundings (school-based OR school).

### 2.3. Eligibility Criteria

This study’s strategy was designed around the PICOS question format with the inclusion criteria (1) (P—Population): participants were children and adolescents aged 6 and 19 who were overweight or obese and without any contraindication to exercise; (2) (I—Intervention): studies with PA intervention programs implemented in the school setting; (3) (C—Comparator): the control group of each study did not participate in any structured PA interventions or weight/obesity management programs; (4) (O—Outcome): studies reported outcomes of one or more cardiometabolic risk factors, with five main aspects including body composition, cardiorespiratory fitness, blood pressure, blood lipids, and blood glucose; (5) (S—Study type): intervention studies with a comparator/control group, and articles were published in a peer-reviewed journal in English.

Exclusion criteria were as follows: (1) observational studies, qualitative studies, reviews, books, dissertations, conference proceedings, commentaries, and studies without full text; (2) studies conducted only with healthy children or do not have subgroups with overweight or obese symptoms; (3) studies mentioned PA intervention but conducted with multiple simultaneous interventions (e.g., cognitive behavior and parental involvement); (4) studies recruited participants in local primary and secondary schools, but the intervention site was outside of the schools; and (5) studies that do not specifically describe the intervention planning and control approach.

### 2.4. Study Selection Process

After conducting the initial search and removing all duplicates, two reviewers were trained to screen titles/abstracts independently and full text for inclusion according to the PICOS strategy. Subsequently, studies that demonstrated potential eligibility in the previous phase were thoroughly reviewed in full, along with those for which eligibility could not be confirmed solely through title and abstract due to insufficient information. In instances of disagreement between the first two reviewers, an additional contributor (FL) was consulted to reach a final determination regarding eligibility.

### 2.5. Data Extraction Process

All data from the included records were extracted by one reviewer (DM) and double-checked by a second reviewer (BL). For each study, we coded the following bibliographic information: (1) first author’s name, (2) publication year, (3) article title. Then, the following details from each study were coded by (1) methodological design, (2) participant characteristics (age, sample size, and the number of boys and girls), (3) characteristics of the intervention protocols, such as the types of PA performed, (4) the frequency per week, the duration of each session, the total duration of the intervention, and whether follow-up assessments were conducted, (5) measures and outcomes of cardiometabolic risk factors, and (6) main findings (effectiveness of the school-based PA intervention on the cardiometabolic risk outcomes). Finally, data from the assessments employed for each outcome were extracted and tabulated in Microsoft Excel software 2021, Version 16.92 (24120731), Office LTSC Standard for Mac 2021.

### 2.6. Quality Assessment Process

Two reviewers independently rated the methodological quality of all the included studies. In case of disagreement in the final evaluation, the third reviewer (FL) performed a new assessment, followed by a discussion for a consensus. In this study, the risk of bias version 2 (RoB 2) was used to evaluate all included randomized controlled trials (RCTs) [26], and the risk of bias in non-randomized studies of interventions (ROBINS-I) was used to evaluate non-randomized controlled studies (non-RCTs) [27].

### 2.7. Data Analysis Process

Stata17.0 software was used for statistical processing. Outcomes of cardiovascular metabolic risk factors were classified and used to perform a meta-analysis, respectively. The aggregate effect estimate was obtained by calculating the score of data change before and after each group, its standard deviation (standard error or 95% confidence interval), and the number of participants in each group. The effect sizes were quantified using Hedges values, and the analyses were conducted utilizing random-effects models. In addition, 0.2 ≤ Hedges’ g < 0.5, 0.5 ≤ Hedges’ g < 0.8, and Hedges’ g ≥ 0.8 were determined to be small, medium, and large effect amounts [28]. Statistical heterogeneity was evaluated by Cochran’s Q statistic and the I^2^ inconsistency test, with *p*-value < 0.1 and I^2^ > 75% considered indicators of high heterogeneity [29]. Egger’s test was used to detect a publication bias, and significance is established with a *p*-value < 0.05.

## 3. Results

### 3.1. Study Selection

**Figure 1:**
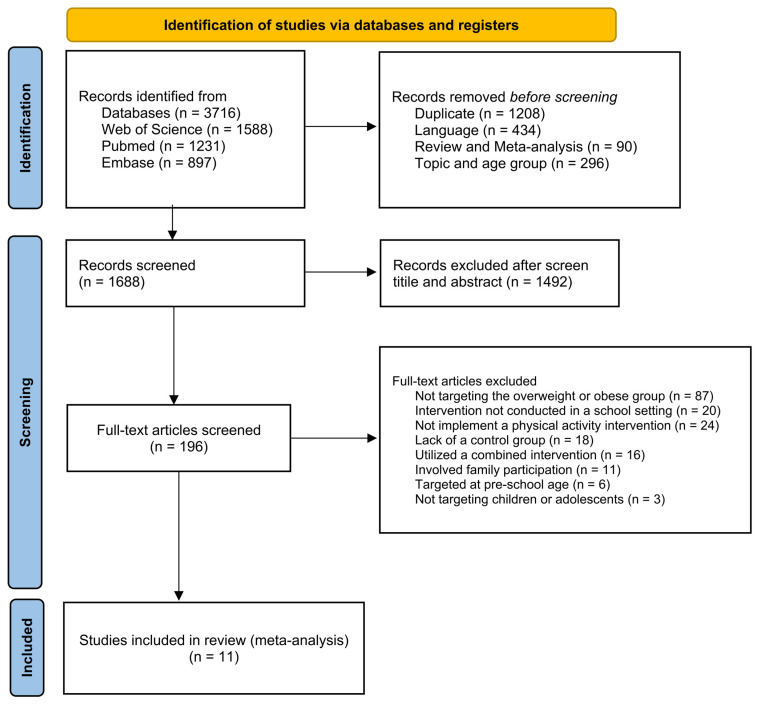
Prisma flow chart.

Using the search protocol, there were 3716 potentially eligible articles. After removing duplications and screening for titles, abstracts, and full texts, eleven records were included in this systematic review and meta-analysis. The flowchart for the selection process is shown in Figure 1.

### 3.2. Study Characteristics

The characteristics of the 11 included studies conducted in 8 countries, consisting of 15 trials, are summarized in Table 1. Eight studies were RCTs, and three were non-RCTs. The majority of the included studies were published within the last three years [22,23,24,30,31,32,33]. Only two studies reported long-term follow-up data [31,33]. More than half of the studies (*n* = 6, 54.54%) reported participant adherence/satisfaction with positive engagement demonstrated [23,30,31,32,34,35]. None of the control group received any PA interventions, and they all maintained their usual daily school-life behaviors. All included trials reported statistical significance for at least one cardiovascular risk indicator.

### 3.3. Participant Characteristics

A total of 963 children and adolescents aged 7 to 18 years were included in the studies (517 in the intervention group and 446 in the control group). The gender distribution was approximately 51.07% boys and 48.93% girls. All studies were focused exclusively on students who were overweight or obese, with individual study sample sizes ranging from 30 to 140 participants. Two studies from China specifically reported the effects of school-based PA interventions in adolescents with intellectual disabilities [31,32]. The majority included studies (*n* = 10, 90.91%) that explicitly reported no significant differences between intervention and control groups at baseline, i.e., sample characteristics were similar across groups at baseline. Half of the included studies (*n* = 6, 54.55%) reported monitoring lifestyle variables such as energy intake and sleep duration, with no significant differences found between the intervention and control groups during the intervention period [22,23,30,31,32,34], while the remaining studies did not measure these variables.

### 3.4. Intervention Characteristics

Interventions in the selected studies were conducted in school settings and supported by school schedules, venue resources, and faculty members. In terms of intensity, the vast majority of trials (*n* = 12, 80.00%) used moderate and above exercise intensity, with HRmax > 70% and HRR > 40%, while three trials utilized a combination exercise of low and moderate or high intensity [30,32]. In terms of single duration, most of the interventions (*n* = 11, 73.33%) lasted 30 minutes or more, with six trials lasting an hour [24,30,34]. In terms of frequency, intervention in all included studies was conducted at least twice per week; three studies conducted interventions every working day [24,36], with one of them conducted twice a day [35]. Overall, the duration of interventions ranged from a minimum of 6 weeks to a maximum of 48 weeks, with seven studies lasting 24 weeks or longer [24,30,32,33,34,35,36].

#### 3.4.1. Type of Intervention

Intervention in three studies used exercise training, including sprint interval training (SIT), high-intensity interval training (HIIT), moderate-intensity continuous training (MICT), and HIIT games [22,23,37]. Intensity may be prioritized over diverse content design among these interventions. However, one study used a combination of games and HIIT [37], which aimed to increase the motivation, enjoyment, and persistence of participants and to ensure that children engaged in each game with high intensity.

Intervention in eight studies used structured movement. This category focuses on aerobic games, resistance exercise, less competition, and an emphasis on increasing exercise and promoting lifestyle changes. Four of these carry creative designs for the traditional physical education (PE) curriculum structure [30,31,32,36]. Unlike the traditional PE class, these interventions feature special content and intensity and focus on enhancing specific health and fitness outcomes among overweight and obese students.

#### 3.4.2. Time Point of Intervention

A total of five studies implemented PA interventions during PE class. One study utilized the cool-down period of PE class to conduct SIT and HIIT [22]. Other programs redesign the entire PE class to focus on fun and engaging activities and promote higher levels of physical exertion [22,30,31,32,36].

A total of six studies carried out interventions during recess or extracurricular time. This category includes interventions that take place outside of regular class hours, which aim to complement the school’s routine activities. One study organized ten-minute games and exercises during class break time [35]. Two studies used activity-class time during school hours [23,37]; others utilized the after-school programs [24,33,34].

**Table 1:** General characteristics of the selected studies.

Study			Sample				Intervention							Main Findings
Author (Year)	Country	Design	Age (Years) (M *±* SD/Rng)	Sex	*n* (C)	*n* (T)	Type	Time Point	Intensity	HRmax/HRR	Min per Class/Section	Days per Week	Total Weeks	
Gonzalez-Galvez et al. (2024) [22]	Spain	RCT	12.51 ± 0.75	Boy/Girl: 18/14	12	SIT group: 9 HIIT group: 11	Exercise training	cool-down period of the PE class	SIT group: h HIIT group: h	SIT group: 90–95% HRmax HIIT group: 80–85% HRmax	12	2	8	SIT group: FM%↓, SBP↔, DBP↔, VO_2_max↔ HIIT group: FM%↓, SBP↓, DBP↓, VO_2_max↑
Meng et al. (2022) [23]	China	RCT	11.2 ± 0.7	Boy: 36	13	HIIT group: 12 MICT group: 11	Exercise training	activity class	HIIT group: h MICT group: m	HIIT group: 80~90% HRmax MICT group: 70% HRmax	HIIT: 11 MICT: 30	3	12	HIIT group: BMI↓, VO_2_max↑, LDL-C↓, HOMA-IR↓ MICT group: BMI↓, VO_2_max↑, BF%↓, HOMA-IR↓
Ponnambalam et al. (2022) [24]	India	RCT	11–14	NA	140	140	Structured movement	Staying after school	m, h	NA	60	5	36	BMI↓, WHtR↔
Machado et al. (2022) [33]	Brazil	non-RCT	10.3 ± 1.8	Boy/Girl: 14/21	15	20	Structured movement	after school	m, h	NA	50	2	24	z-BMI↓, WHtR↓, SBP↓, DBP↓, FBG↓,
Lambrick et al. (2016) [37]	New Zealand	RCT	8–10	Boy/Girl: 17/12	14	15	Exercise training	Extracurricular time	h	86% HRmax	40	2	6	VO_2_max↑, WC↓
Seabra et al. (2016) [34]	Portugal	non-RCT	8–12	Boy: 59	30	29	Structured movement	Staying after school	m, h	70–80% HRmax	60–90	3	24	z-BMI↓, WC↓, TG↓, VO_2_max↑
Carrel et al. (2005) [36]	America	RCT	12 ± 0.5	Boy/Girl: 26/24	23	27	Structured movement	PE class	m	NA	45	5	36	BF%↓, VO_2_max↑, FBG↓
Wang et al. (2015) [35]	China	non-RCT	7–12	Boy/Girl: 82/170	136	116	Structured movement	class break	m	NA	10	10(twice a day)	48	BF%↓, SBP↓, DBP↓
Wang et al. (2022) [31]	China	RCT	14.17 ± 0.45/12–18	Boy/Girl: 22/8	15	15	Structured movement	PE class	m, h	40~70% HRR	60	2	12	BMI↓, SBP↔, DBP↔
Yu et al. (2022) [32]	China	RCT	14.98 ± 1.63/12–18	Boy/Girl: 45/16	22	39	Structured movement	PE class	l, m	30~60% HRR	45	2	36	BMI↓, z-BMI↓, BF%↓, WC↓, WHtR↓
Gonzalez-Ruiz et al. (2021) [30]	Colombia	RCT	13.49 ± 1.65/11–17	Boy/Girl: 31/68	26	HIPE group: 24 LIPE group: 24 PLUS group: 25	Structured movement	PE class	HIPE group: h LIPE group: l, m PLUS group: l, m, h	HIPE group: >70%HRmax LIPE group: 50~70%HRmax PLUS group: >50% HRmax	60	2	24	HIPE group: z-BMI↓, BF%↓, TC↓, LDL-C↓ LIPE group: BMI↓, BF%↓, TC↔, HDL-C↑ LDL-C↔ PLUS group: TG↓, BF%↔, TC↔, LDL-C↔

Notes: C, control group; T, intervention group; h, high intensity; m, moderate intensity; l, low intensity; HRmax, maximum heart rate; HRR, heart rate reserve; FM%, fat mass percentage; BMI, body mass index; z-BMI, body mass index z-score; BF%, body fat percentage; WC, waist circumference; WHtR, waist-to-height ratio; VO_2_max, maximal oxygen consumption; SBP, systolic blood pressure; DBP, diastolic blood pressure; HDL-C, high-density lipoprotein cholesterol; LDL-C, low-density lipoprotein cholesterol; TG, triglycerides; TC, total cholesterol; FBG, fasting blood glucose; BI, blood insulin; HOMA-IR, homeostatic model assessment-insulin resistance; SIT, sprint interval training; HIIT, high-intensity interval training; MICT, moderate-intensity continuous training; HIPE, high-intensity physical education; LIPE, low-to-moderate intensity physical education; PLUS, combined HIPE and LIPE; “↑” indicates a significant increase, “↓” indicates a significant decrease, and “↔” indicates no significant change.

### 3.5. Risk of Bias

The general scores in the RoB 2 and ROBINS-I are shown in Supplementary Tables S1 and S2. Four studies had a low overall bias among the eight RCTs evaluated in RoB 2. By domain, the majority of RCTs (75%) have some concern in terms of deviations from intended interventions, with the application of participant blinding not fully feasible due to the inherent nature of the interventions; 37.5% of the included RCTs have shortcomings in the randomization process; all of the RCTs have a low risk of missing outcome data, with specifically reported reasons for participant withdrawal; 87.5% of the included RCTs have a low risk of measurement of the outcome and selection of the reported result. The three non-RCTs evaluated in ROBINS-I showed a moderate overall risk of bias, with the terms “selection of participants into the study” and “measurement of outcomes” having some concerns.

### 3.6. Meta-Analyses Results

The results of the meta-analysis of 15 outcome indicators in the present study are shown in Table 2, and the forest plots of the effect size of each are shown in the Supplementary Figures (see Figures S1–S15).

#### 3.6.1. Effects of School-Based PA Interventions on Body Composition

Data on BMI were available from 9 studies with 11 comparisons, including 829 overweight or obese participants (intervention and control). Pooled results showed that school-based PA interventions were not statistically associated with changes in BMI in comparison with the control group (Hedges’ g = −0.42, 95% CI = [−0.92, 0.09], *p* = 0.10; I^2^ = 90.54%) (see Figure S1).

Data on z-BMI were available from 4 studies with 6 comparisons, including 240 overweight or obese participants (intervention and control). Pooled results showed that school-based PA interventions were not statistically associated with changes in z-BMI in comparison with the control group (Hedges’ g = −0.11, 95% CI = [−0.34, 0.12], *p* = 0.34; I^2^ = 0.00%) (see Figure S2).

Data on BF% were available from 8 studies with 11 comparisons, including 616 overweight or obese participants (intervention and control). Pooled results showed that school-based PA interventions were statistically associated with changes in BF% in comparison with the control group (Hedges’ g = −0.43, 95% CI = [−0.73, −0.13], *p* < 0.01; I^2^ = 67.67%) (see Figure S3).

Data on WC were available from 7 studies with 10 comparisons, including 566 overweight or obese participants (intervention and control). Pooled results showed that school-based PA interventions were not statistically associated with changes in WC in comparison with the control group (Hedges’ g = −0.19, 95% CI = [−0.37, −0.00], *p* = 0.05; I^2^ = 15.55%) (see Figure S4).

Data on WHtR were available from 4 studies with 4 comparisons, including 141 overweight or obese participants (intervention and control). Pooled results showed that school-based PA interventions were not statistically associated with changes in WHtR in comparison with the control group (Hedges’ g = −0.26, 95% CI = [−0.60, 0.08], *p* = 0.13; I^2^ = 0.00%) (see Figure S5).

#### 3.6.2. Effects of School-Based PA Interventions on Cardiorespiratory Fitness

Data on VO_2_max were available from 5 studies with 7 comparisons, including 206 overweight or obese participants (intervention and control). Pooled results showed that school-based PA interventions were not statistically associated with changes in VO_2_max in comparison with the control group (Hedges’ g = 0.63, 95% CI = [−0.03, 1.30], *p* = 0.06; I^2^ = 82.22%) (see Figure S6).

#### 3.6.3. Effects of School-Based PA Interventions on Blood Pressure

Data on SBP were available from 7 studies with 11 comparisons, including 529 overweight or obese participants (intervention and control). Pooled results showed that school-based PA interventions were not statistically associated with changes in SBP in comparison with the control group (Hedges’ g = −0.05, 95% CI = [−0.43, 0.32], *p* = 0.77; I^2^ = 75.81%) (see Figure S7).

Data on DBP were available from 7 studies with 11 comparisons, including 529 overweight or obese participants (intervention and control). Pooled results showed that school-based PA interventions were statistically associated with changes in DBP in comparison with the control group (Hedges’ g = −0.27, 95% CI = [−0.50, −0.04], *p* = 0.02; I^2^ = 36.23%) (see Figure S8).

#### 3.6.4. Effects of School-Based PA Interventions on Blood Lipids

Data on HDL-C were available from 5 studies with 8 comparisons, including 467 overweight or obese participants (intervention and control). Pooled results showed that school-based PA interventions were not statistically associated with changes in HDL-C in comparison with the control group (Hedges’ g = 0.15, 95% CI = [−0.12, 0.42], *p* = 0.28; I^2^ = 46.27%) (see Figure S9).

Data on LDL-C were available from 5 studies with 8 comparisons, including 467 overweight or obese participants (intervention and control). Pooled results showed that school-based PA interventions were not statistically associated with changes in LDL-C in comparison with the control group (Hedges’ g = −0.26, 95% CI = [−0.62, 0.11], *p* = 0.16; I^2^ = 70.07%) (see Figure S10).

Data on TG were available from 5 studies with 8 comparisons, including 467 overweight or obese participants (intervention and control). Pooled results showed that school-based PA interventions were statistically associated with changes in TG in comparison with the control group (Hedges’ g = −0.38, 95% CI = [−0.63, −0.13], *p* < 0.01; I^2^ = 36.23%) (see Figure S11).

Data on TC were available from 4 studies with 7 comparisons, including 446 overweight or obese participants (intervention and control). Pooled results showed that school-based PA interventions were not statistically associated with changes in TC in comparison with the control group (Hedges’ g = −0.22, 95% CI = [−0.67, 0.23], *p* = 0.33; I^2^ = 79.94%) (see Figure S12).

#### 3.6.5. Effects of School-Based PA Interventions on Blood Glucose

Data on FBG were available from 6 studies with 9 comparisons, including 517 overweight or obese participants (intervention and control). Pooled results showed that school-based PA interventions were statistically associated with changes in FBG in comparison with the control group (Hedges’ g = −0.60, 95% CI = [−0.92, −0.28], *p* < 0.01; I^2^ = 65.03%) (see Figure S13).

Data on BI were available from 5 studies with 8 comparisons, including 265 overweight or obese participants (intervention and control). Pooled results showed that school-based PA interventions were statistically associated with changes in BI in comparison with the control group (Hedges’ g = −0.62, 95% CI = [−0.84, −0.40], *p* < 0.01; I^2^ = 0.00%) (see Figure S14).

Data on HOMA-IR were available from 3 studies with 6 comparisons, including 194 overweight or obese participants (intervention and control). Pooled results showed that school-based PA interventions were statistically associated with changes in HOMA-IR in comparison with the control group (Hedges’ g = −0.58, 95% CI = [−0.95, −0.21], *p* < 0.01; I^2^ = 51.82%) (see Figure S15).

## 4. Discussion

In this study, we present the review of the evidence from 11 studies with 15 control trials, evaluating the effects of school-based PA interventions in reducing cardiometabolic risk factors in overweight or obese children and adolescents from 7 to 18 years of age. The findings of our study demonstrated the following: During the 6 weeks to 12 months intervention period with twice a week or more, (1) significant reductions were noted in BF%, DBP, TG, FBG, BI, and HOMA-IR; and (2) no significant changes were observed in BMI, z-BMI, WC, WHtR, VO_2_max, SBP, HDL-C, LDL-C, and TC.

Overall, in the present study, we found that school-based PA interventions have significant efficacy in improving blood glucose levels among overweight or obese children and adolescents while exhibiting relatively limited effectiveness on body composition, cardiorespiratory fitness, and blood lipids. To some extent, interventions may not be of sufficient duration and intensity to have significant effects on these parameters in this population and may be limited by multiple external factors. A recent systematic review and meta-analysis revealed that school-based PA interventions have a small positive impact on students’ total PA level but do not influence their leisure-time activity behaviors, with outcomes significantly influenced by theoretical frameworks, external expert and family involvement, and staff training [38]. Additionally, existing studies have focused on short-term interventions, and most have not followed up over time [39], so the long-term sustainability and potential delayed effects of these interventions remain inadequately assessed due to the lack of robust longitudinal data. However, these strategies may be important and feasible in the long term for improving cardiovascular health outcomes in overweight or obese individuals within school routines [40], despite the impact appearing to be moderate. We thus suggest that future research should conduct long-term interventions and carry out ongoing follow-up studies to explore the sustained effects of each risk factor.

### 4.1. Body Composition

This study showed that school-based PA interventions significantly reduced BF% in overweight or obese children and adolescents, emphasizing the need to enhance their daily PA levels to reduce excess adiposity. In particular, our study shows no significant effect on BMI, accompanied by high heterogeneity. This finding differs from that of a previous meta-analysis conducted in a similar population, which showed that exercise interventions significantly reduce BMI with a moderate effect [19]. A plausible explanation for this inconsistency could lie in the nature of the interventions among the studies included in the present study that varied considerably, with exercise training potentially exerting greater efficacy. Our study encompassed BMI-related parameters from all included studies. Seven studies exhibited statistically significant reductions in BMI or z-BMI [23,24,30,31,32,33,34]; however, a study of 116 overweight or obese children doing two daily “Happy 10 Minutes” activities found no significant improvement, possibly due to insufficient exercise intensity [35]. Also, no significant effect on z-BMI was found, with low heterogeneity, similar to previous studies. A Cochrane review showed that interventions resulted in minimal improvement in the z-BMI of 6–12-year-olds [41], and another meta-analysis indicated that gains were only temporary without sustained interventions [42]. For WC and WHtR outcomes, no significant differences were found in our study, consistent with a previous meta-analysis study among specific-obese youth [13]. In contrast, a meta-analysis of the general children population demonstrated positive effects [43], but another systematic review suggested that PA interventions may not be particularly effective in specific-disability children and adolescents [44]. This may be attributable to the physiological and psychological heterogeneity within the research population. Evidence showed that metabolic abnormalities in obese teens can lead to significant accumulation of abdominal fat [45]. Short-term interventions are often insufficient to achieve noticeable results, and higher stress levels, depressive symptoms, and lower resilience may further complicate the effectiveness of interventions [46].

### 4.2. Cardiorespiratory Fitness

VO_2_max has consistently been identified as a strong predictor of adverse health outcomes such as cardiovascular disease and all-cause mortality [47]. In the present study, no significant improvement in VO_2_max was observed, with considerable heterogeneity possibly due to the type of PA intervention. The results of our study differ from previous studies, as a systematic review demonstrated that HIIT training programs exceeding 10 weeks in duration can significantly enhance VO_2_max in children and adolescents with overweight or obesity by approximately 10% and outperforming traditional moderate-intensity continuous training [48]. To some extent, this finding in the present study may also be attributed to the inherent characteristics of overweight and obese groups, which typically exhibit lower cardiorespiratory fitness. Studies indicated that being overweight or obese imposes an additional metabolic burden on the body, requiring increased cardiac and pulmonary efforts to meet physiological demands, which can lead to fatigue during PA and thus limit the efficacy of exercise in enhancing cardiorespiratory function [49]. Specifically, during exercise, they experience more rapid increases in heart and respiratory rates, necessitating harder work from the heart and lungs to meet the body’s oxygen and energy demands. Notably, while VO_2_max can be significantly enhanced through exercise training, the intensity of the exercise is not a determining factor. Previous studies found that significant gains in VO_2_max can be achieved even at lower intensities by increasing training volume or adjusting training cycles [50,51]. Thus, future research should prioritize long-term PA interventions among this population and identify the barriers and facilitators of adherence.

### 4.3. Blood Pressure

Our study indicates that school-based PA interventions significantly reduce overweight or obese children and adolescents in DBP with lower heterogeneity but do not notably affect SBP. These findings emphasize the significance of exercise for blood pressure reduction, similar to previous research demonstrating that a significant effect is observed only in DBP [52,53]. For instance, a meta-analysis of school-based PA interventions targeting normal-weight children aged 3 to 12 years demonstrated a significant effect size for DBP level (Hedges’ g = −0.21; 95% CI = [−0.42 to −0.01], *p* = 0.04) and presumed the differential effects observed on DBP and SBP might be attributed to the neurohumoral, vascular, and structural adaptations that occur in response to PA [43]. However, even though PA interventions have been considered one of the key factors in reducing blood pressure, studies suggest that PA interventions alone may not provide enough evidence of blood pressure reduction, particularly in normal children and adolescents [54]. Thus, the reduction of SBP and DBP in this group is still uncertain and requires further evidence. Considering that overweight and obese children and adolescents are at high risk of hypertension, future studies should further clarify the mechanisms of antihypertensive PA interventions for this population.

### 4.4. Blood Lipids

Our study found that school-based PA interventions can significantly reduce TG levels in overweight or obese children and adolescents, while there are positive results for HDL-C, LDL-C, and TC without statistical significance. These findings indicate that PA interventions may positively improve obese children and adolescents’ levels of blood lipids and reduce the risk of hyperlipidemia. Hyperlipidemia is a common metabolic disorder characterized by elevated plasma TG, TC, and LDL-C, with concomitant reductions in HDL-C [55]. However, the evidence on cholesterol effects is still mixed and needs more evidence. For instance, a Cochrane review reported significant effects in children [52], while another meta-analysis conducted in overweight and obese adolescents found that there was a likely trivial improvement in LDL-C but unclear changes in the levels of TC and HDL-C [19]. For obese children and adolescents, dyslipidemia may be associated with metabolic syndrome, thereby making it difficult to significantly improve cholesterol levels through PA intervention alone, with metabolism influenced by a variety of factors, including genetics, diet, and lifestyle, which may require longer-term interventions or more comprehensive lifestyle changes [56].

### 4.5. Blood Glucose

Importantly, this study found that school-based PA interventions can significantly improve the condition of blood glucose in overweight or obese children and adolescents, including the levels of FBG, BI, and HOMA-IR, with moderate effect size and lower heterogeneity. These findings are similar to previous research that exercise is an important strategy for managing blood glucose levels; by improving insulin sensitivity, direct glucose utilization, increasing muscle mass, and regulating hormone balance, exercise can significantly improve glycemic control and overall metabolic health [57]. Additionally, HIIT may be more effective than traditional moderate-intensity exercise in improving glycemic control [58]. However, an intervention study on aerobic exercise for obese children found that, after 24 weeks of exercise interventions, although there was no significant change in the participant’s blood glucose levels, endothelial function was significantly improved, suggesting that exercise may indirectly affect glucose metabolism through other mechanisms [59].

### 4.6. Limitations and Prospects

We also acknowledge several specific limitations in the present study. First, due to the limited number of available eligible studies, subgroup analyses were not suitable for further exploration in this study, which could have otherwise led to more precise identification of the best options for school PA interventions, including discussions on intervention type and timing; some included studies involved both children and adolescents, and the large age range spanned made it difficult to separate the two groups, potentially affecting the results and introducing heterogeneity. Second, the sample sizes of the trials included in the analyses were generally small, and the number of trials reporting on variables (e.g., z-BMI, WHtR, HOMA-IR, etc.) was limited, which may affect the statistical power and generalizability of the findings. Third, the studies included in the analysis had some risks, and half of the total lacked information on intervention adherence; some of them did not further rigorously control for a range of confounding factors such as diet, sleep, etc., leading to less robust results regarding the effects of PA interventions on cardiovascular risk factors.

Currently, there is increasing prominence of obesity in children and adolescents, while the indicators for assessing cardiometabolic risk in this population are still fragmented. To improve the consistency and comparability of research results, subsequent research should adopt standardized cardiometabolic measurement indicators and methods in this special group. For example, although BMI is the most commonly used index in clinical practice to predict the risk of developing metabolic syndrome, it cannot be used to distinguish between fat-free mass, fat mass, and their distribution [60]. Moreover, research has shown that pediatric patients with BMI-classified obesity do not consistently exhibit metabolic abnormalities such as hypertension, hyperglycemia, dyslipidemia, or insulin resistance [61,62], which suggests the necessity for more comprehensive diagnostic measures.

In short, (1) there is a need for more sample data and high-quality evidence on school-based PA interventions; (2) future research should control for multivariate variables in both intervention and control groups, thereby enabling a more accurate assessment of the effectiveness of PA interventions and facilitating the development of more effective strategies; (3) further exploration is necessary for the adoption of unified and comprehensive indicators that could refine obesity diagnosis, diminishing the risk of misclassification and offering novel perspectives for a global strategy against obesity.

## 5. Conclusions

The present study’s findings indicate that school-based PA interventions can significantly reduce BF%, DBP, TG, FBG, BI, and HOMA-IR in overweight or obese children and adolescents; there were no significant effects on BMI, z-BMI, WC, WHtR, VO_2_max, SBP, HDL-C, LDL-C, and TC. Interventions lasting 6 weeks longer, twice a week or more, can effectively mitigate some cardiovascular risk factors. These findings highlight the importance of school-based PA programs to satisfy this population’s special needs. We recommended that school administrators prioritize the health concerns of overweight or obese students by incorporating effective and targeted PA intervention plans into the school’s daily routines and schedules. However, since lifestyle factors (e.g., diet and sleep) that may influence cardiovascular risk factors were not strictly controlled, our findings should be viewed with caution.

## Figures and Tables

**Table 2:** Summary of meta-analysis results.

Category	Outcome Indicators	Trials (*n*)	Heterogeneity Test Results (I^2^-Value)	Meta-Analysis Results Hedges’ g [95%CI]
Body composition	BMI (kg/m^2^)	11	90.54% **	−0.42 [−0.92, 0.09]
	z-BMI	6	0.00%	−0.11 [−0.34, 0.12]
	BF (%)	11	67.67% **	−0.43 [−0.73, −0.13] **
	WC (cm)	10	15.55%	−0.19 [−0.37, −0.00]
	WHtR	4	0.00%	−0.26 [−0.60, 0.08]
Cardiorespiratory fitness	VO_2_max (mL/kg/min)	7	82.22% **	0.63 [−0.03, 1.30]
Blood pressure	SBP (mmHg)	11	75.81% **	−0.05 [−0.43, 0.32]
	DBP (mmHg)	11	36.23%	−0.27 [−0.50, −0.04] *
Blood lipids	HDL-C (mg/dL)	8	46.27%	0.15 [−0.12, 0.42]
	LDL-C (mg/dL)	8	70.07% **	−0.26 [−0.62, 0.11]
	TG (mg/dL)	8	36.23%	−0.38 [−0.63, −0.13] **
	TC (mg/dL)	7	79.94% **	−0.22 [−0.67, 0.23]
Blood glucose	FBG (mg/dL)	9	65.03% **	−0.60 [−0.92, −0.28] **
	BI (uIU/mL)	8	0.00%	−0.62 [−0.84, −0.40] **
	HOMA-IR	6	51.82%	−0.58 [−0.95, −0.21] **

Notes: BMI, body mass index; z-BMI, body mass index z-score; BF%, body fat percentage; WC, waist circumference; WHtR, waist-to-height ratio; VO_2_max, maximal oxygen consumption; SBP, systolic blood pressure; DBP, diastolic blood pressure; HDL-C, high-density lipoprotein cholesterol; LDL-C, low-density lipoprotein cholesterol; TG, triglycerides; TC, total cholesterol; FBG, fasting blood glucose; BI, blood insulin; HOMA-IR, homeostatic model assessment-insulin resistance; * *p* < 0.05, ** *p* < 0.01.

## Data Availability

The data sets generated and/or analyzed during the current research are available from the corresponding author upon reasonable request.

## Supplementary Materials

The following supporting information can be downloaded at: https://www.mdpi.com/article/10.3390/children12040439/s1. Supplementary Figures. Figure S1. Pooled effect size estimated for BMI; Figure S2. Pooled effect size estimated for z-BMI; Figure S3. Pooled effect size estimated for BF%; Figure S4. Pooled effect size estimated for WC; Figure S5. Pooled effect size estimated for WHtR; Figure S6. Pooled effect size estimated for VO_2_max; Figure S7. Pooled effect size estimated for SBP; Figure S8. Pooled effect size estimated for DBP; Figure S9. Pooled effect size estimated for HDL-C; Figure S10. Pooled effect size estimated for LDL-C; Figure S11. Pooled effect size estimated for TG; Figure S12. Pooled effect size estimated for TC; Figure S13. Pooled effect size estimated for FBG; Figure S14. Pooled effect size estimated for BI; Figure S15. Pooled effect size estimated for HOMA-IR; Figure S16. Risk of Bias in RCTs Assessment Using RoB 2 Tool; Figure S17. Risk of Bias in non-RCTs Assessment Using ROBINS-I Tool; Supplementary Tables. Table S1. RoB 2 general scores for RCTs; Table S2. ROBINS-I general scores for non-RCTs.
