# Supplementary material for: Evaluating the Role of School-Based Physical Activity in Mitigating Cardiometabolic Risk Factors in Children and Adolescents with Overweight or Obesity: A Systematic Review and Meta-Analysis
Source: Children (Basel). 2025 Mar 29;12(4):439. doi: 10.3390/children12040439 (PMC12025731; doi:10.3390/children12040439)
Supplement: Supplementary file 1 [file children-12-00439-s001.zip › Supplementary Figures.pdf]

Supplementary Figures

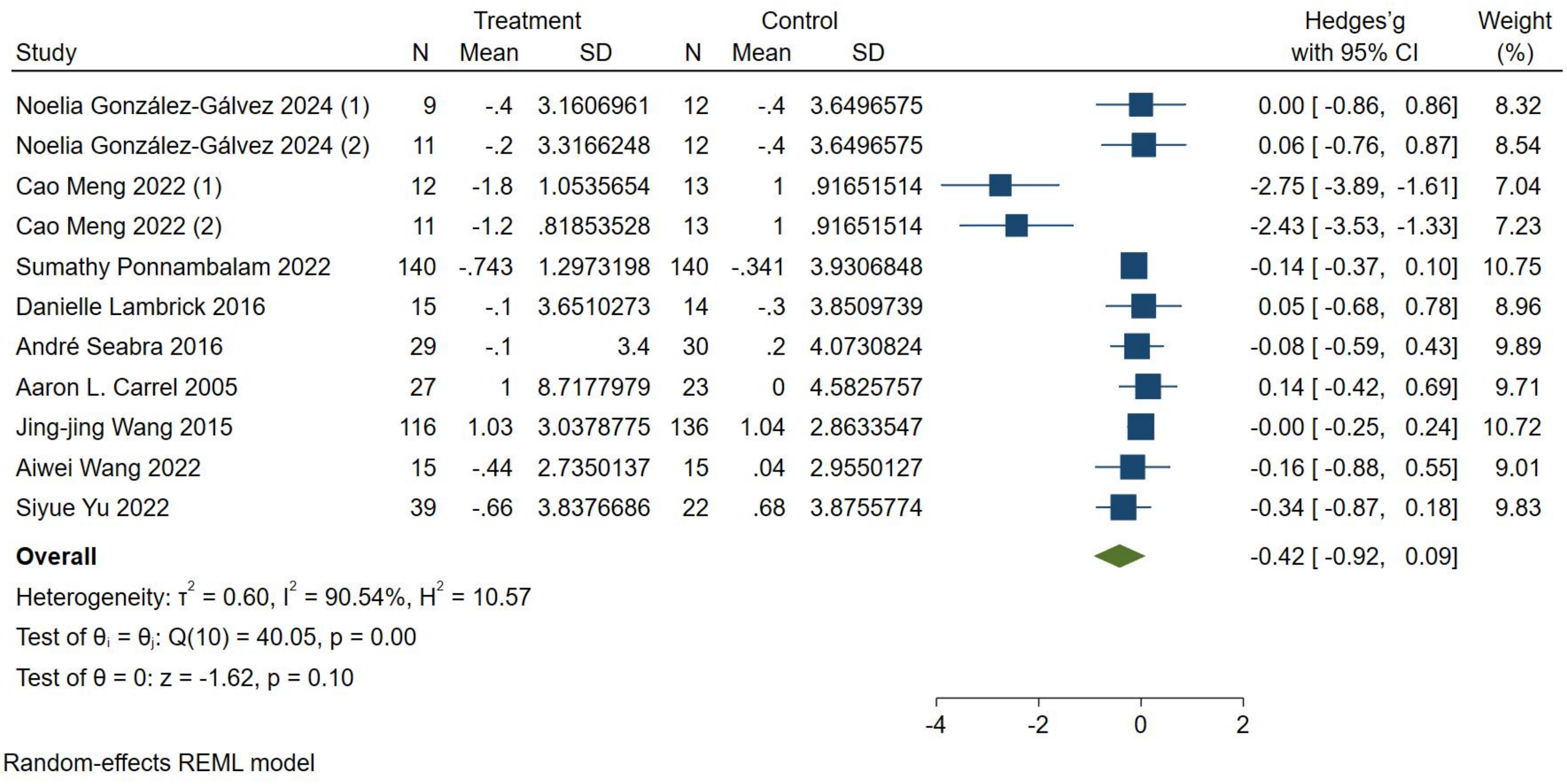

Figure S1 Pooled effect size estimated for BMI

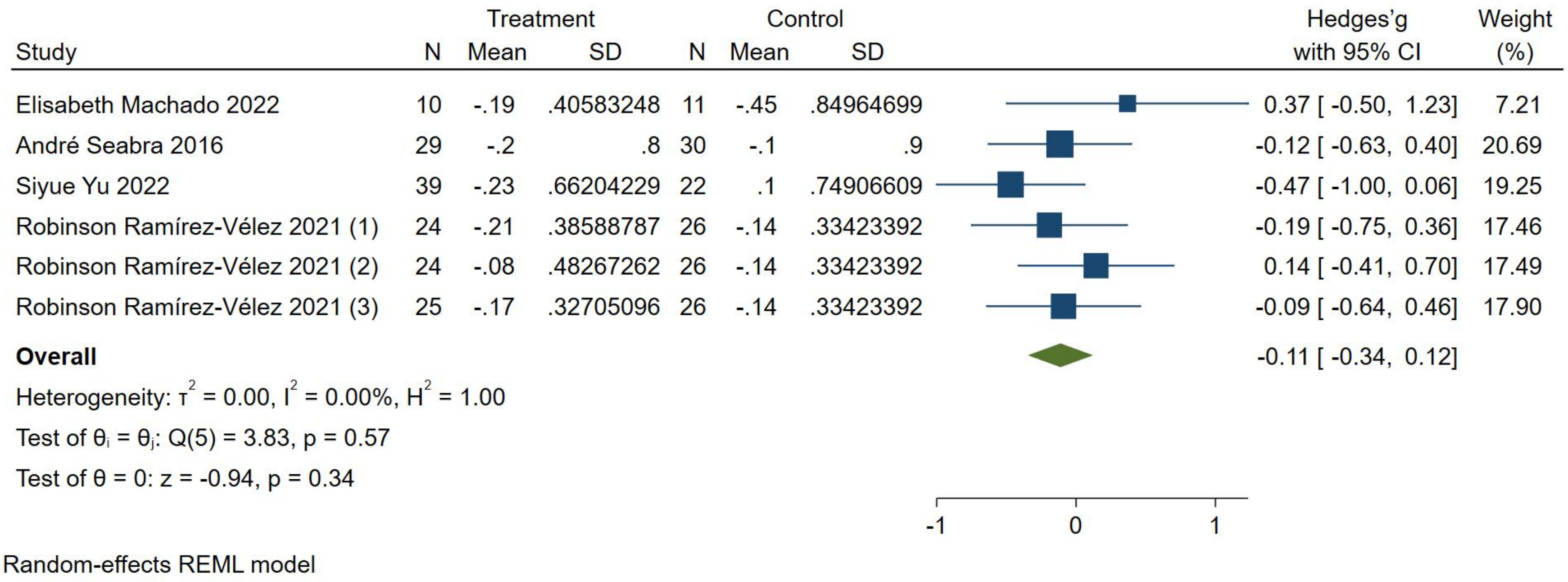

Figure S2 Pooled effect size estimated for z-BMI

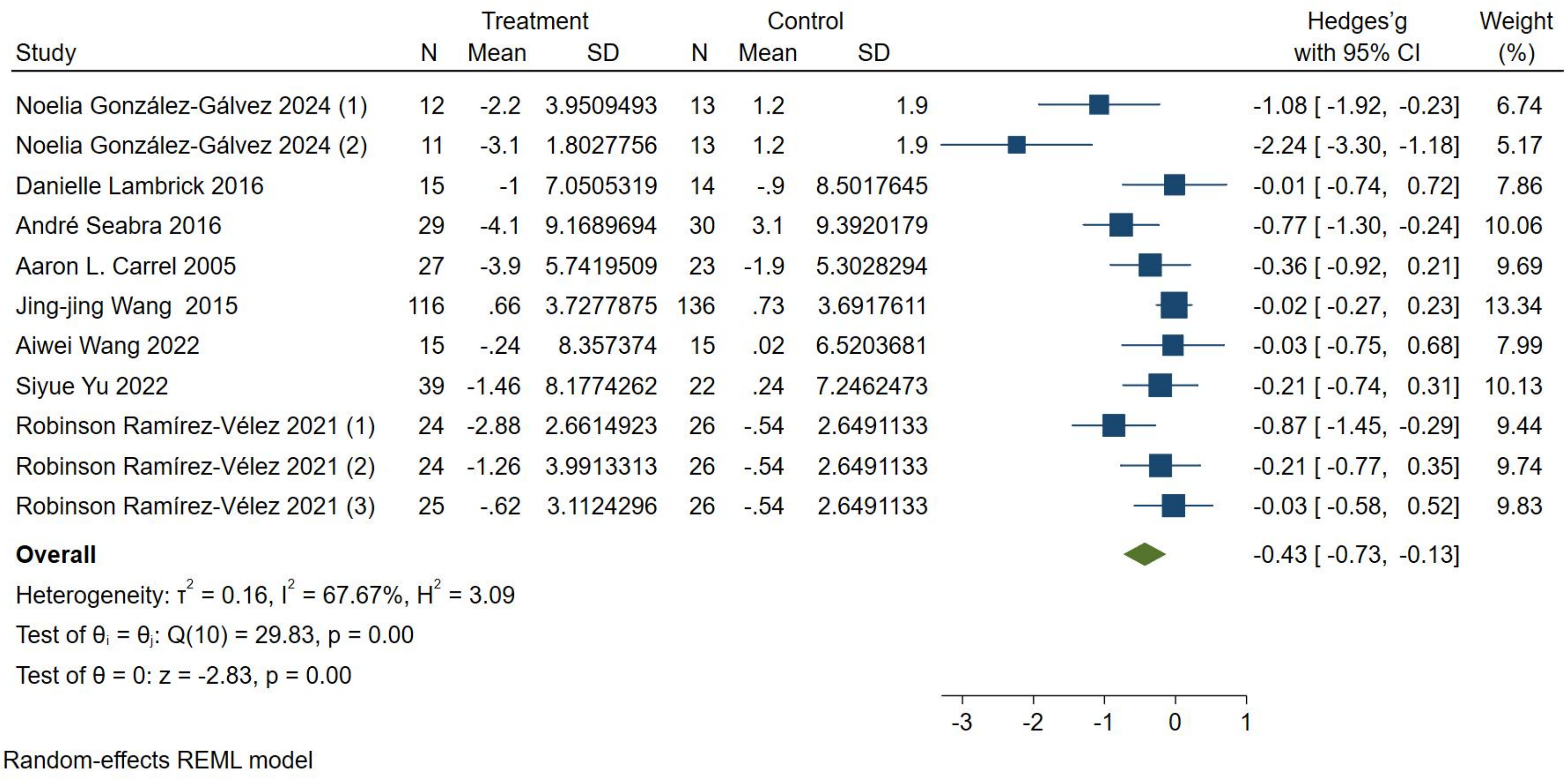

Figure S3 Pooled effect size estimated for BF%

Study	Treatment N	Treatment Mean	Treatment SD	Control N	Control Mean	Control SD	Hedges'g with 95% CI	Weight (%)
Cao Meng 2022 (1)	12	-5	6.8432448	13	2.4	7.1505245	-1.02 [-1.86, -0.18]	4.44
Cao Meng 2022 (2)	11	-.7	6.7535176	13	2.4	7.1505245	-0.43 [-1.24, 0.38]	4.74
Danielle Lambrick 2016	15	-2.3	9.5388679	14	-.2	11.147645	-0.20 [-0.93, 0.53]	5.79
André Seabra 2016	29	-4.3	8.2504545	30	-.2	12.011245	-0.39 [-0.91, 0.12]	10.66
Jing-jing Wang 2015	116	3.26	9.0215132	136	3.06	8.719914	0.02 [-0.23, 0.27]	29.86
Aiwei Wang 2022	15	-.23	5.1983459	15	.02	5.7406533	-0.04 [-0.76, 0.67]	6.00
Siyue Yu 2022	39	-2.01	10.639601	22	1.76	10.704149	-0.35 [-0.88, 0.18]	10.29
Robinson Ramírez-Vélez 2021 (1)	24	-.26	7.1603638	26	.51	6.2266541	-0.11 [-0.67, 0.44]	9.40
Robinson Ramírez-Vélez 2021 (2)	24	1.34	7.3175774	26	.51	6.2266541	0.12 [-0.43, 0.68]	9.40
Robinson Ramírez-Vélez 2021 (3)	25	-2.21	7.3646221	26	.51	6.2266541	-0.39 [-0.95, 0.16]	9.42
**Overall**							-0.19 [-0.37, -0.00]	

Heterogeneity: $\tau^2 = 0.01$, $I^2 = 15.55\%$, $H^2 = 1.18$

Test of $\theta_i = \theta_j$: Q(9) = 9.58, p = 0.39

Test of $\theta = 0$: z = -1.99, p = 0.05

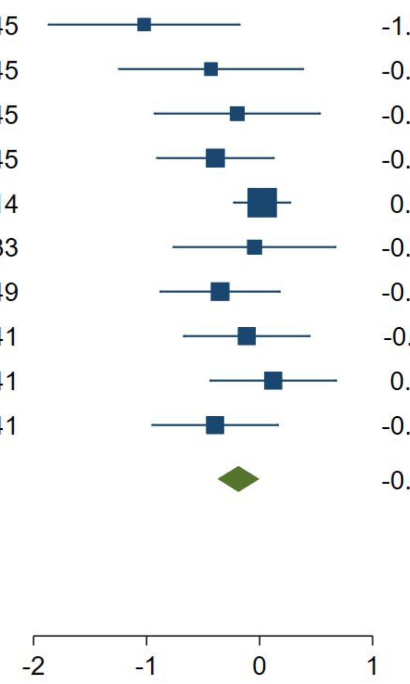

Random-effects REML model

Figure S4 Pooled effect size estimated for WC

Study	Treatment N	Treatment Mean	Treatment SD	Control N	Control Mean	Control SD	Hedges'g with 95% CI	Weight (%)
Elisabeth Machado 2022	10	-.04	.05723635	11	-.02	.04358899	-0.38 [-1.25, 0.49]	15.21
Danielle Lambrick 2016	15	-.01	.06557439	14	0	.08544004	-0.13 [-0.86, 0.60]	21.44
Aiwei Wang 2022	15	-.01	.04	15	0	.05	-0.21 [-0.93, 0.50]	22.11
Siyue Yu 2022	39	-.01	.05567764	22	.01	.07549834	-0.31 [-0.84, 0.21]	41.25
**Overall**							-0.26 [-0.60, 0.08]	

Heterogeneity: $\tau^2 = 0.00$, $I^2 = 0.00\%$, $H^2 = 1.00$

Test of $\theta_i = \theta_j$: Q(3) = 0.25, p = 0.97

Test of $\theta = 0$: z = -1.52, p = 0.13

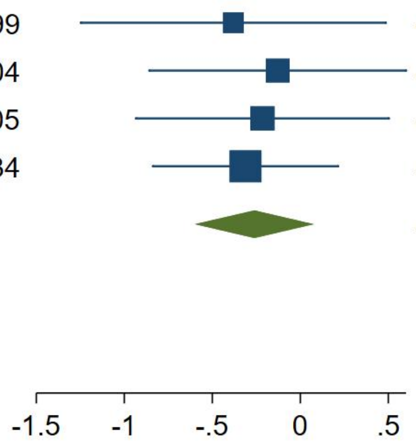

Random-effects REML model

Figure S5 Pooled effect size estimated for WHtR

Study	Treatment N	Treatment Mean	Treatment SD	Control N	Control Mean	Control SD	Hedges'g with 95% CI	Weight (%)
Noelia González-Gálvez 2024 (1)	9	1	3.3406586	12	0	3.6496575	0.27 [-0.60, 1.14]	13.74
Noelia González-Gálvez 2024 (2)	11	2.2	3.6932371	12	0	3.6496575	0.58 [-0.26, 1.42]	13.97
Cao Meng 2022 (1)	12	6.1	2.2869193	13	-.2	2.5159491	2.53 [1.43, 3.62]	12.10
Cao Meng 2022 (2)	11	3	1.9	13	-.2	2.5159491	1.37 [0.46, 2.28]	13.46
Danielle Lambrick 2016	15	-1.5	5.3702886	14	1.8	6.3379808	-0.55 [-1.29, 0.20]	14.65
André Seabra 2016	29	5	8.5539465	30	2.1	12.868955	0.26 [-0.25, 0.77]	16.19
Aaron L. Carrel 2005	27	3	5.5677644	23	.7	5.5677644	0.41 [-0.16, 0.97]	15.88
**Overall**							0.63 [-0.03, 1.30]	

Heterogeneity: $\tau^2 = 0.65$, $I^2 = 82.22\%$, $H^2 = 5.63$

Test of $\theta_i = \theta_j$: Q(6) = 25.48, p = 0.00

Test of $\theta = 0$: z = 1.86, p = 0.06

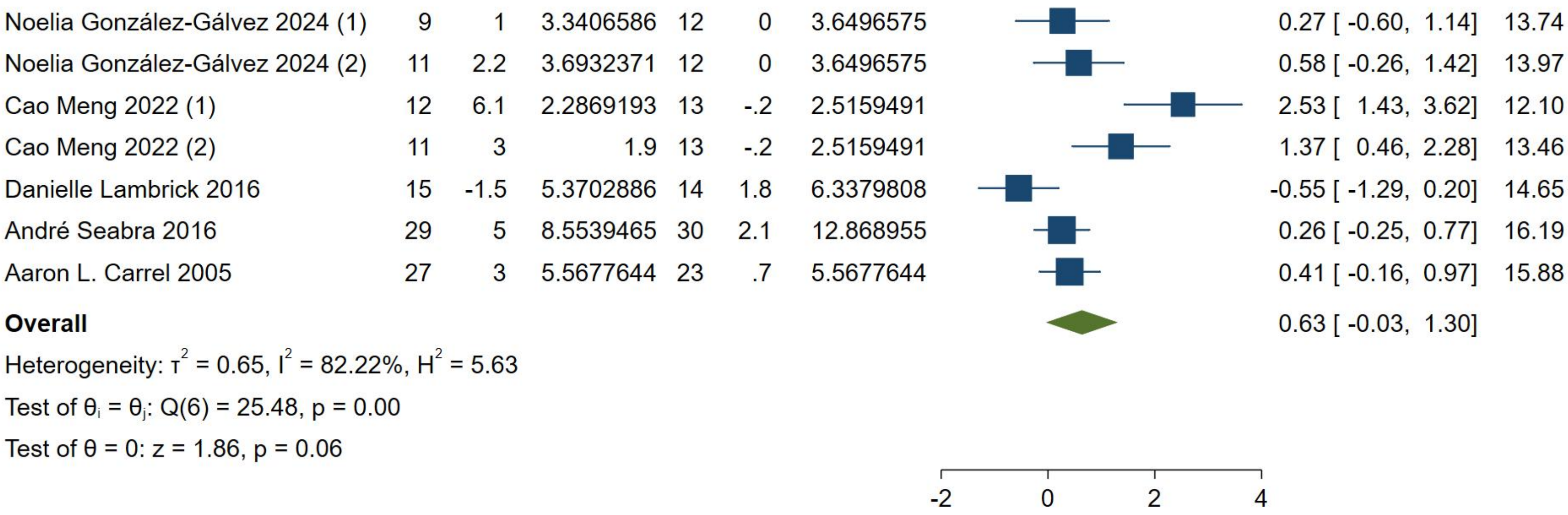

Random-effects REML model

Figure S6 Pooled effect size estimated for VO2max

Study	Treatment N	Treatment Mean	Treatment SD	Control N	Control Mean	Control SD	Hedges'g with 95% CI	Weight (%)
Noelia González-Gálvez 2024 (1)	9	3.1	17.707625	12	-1.8	20.447005	0.24 [-0.62, 1.11]	7.63
Noelia González-Gálvez 2024 (2)	11	-11.8	17.559328	12	-1.8	20.447005	-0.50 [-1.34, 0.33]	7.89
Cao Meng 2022 (1)	12	-5	6.244998	13	1	4.5825757	-1.07 [-1.91, -0.22]	7.78
Cao Meng 2022 (2)	11	-5	6.5574385	13	1	4.5825757	-1.04 [-1.91, -0.18]	7.65
Elisabeth Machado 2022	10	-12.8	6.6302338	11	-3.6	13.036871	-0.84 [-1.74, 0.06]	7.39
André Seabra 2016	29	4.9	10.779147	30	1.4	13.309019	0.28 [-0.23, 0.80]	10.46
Jing-jing Wang 2015	116	5.1	10.950342	136	1.6	9.6534968	0.34 [0.09, 0.59]	12.34
Aiwei Wang 2022	15	-6.8	12.343269	15	-.53	16.35033	-0.42 [-1.15, 0.30]	8.73
Robinson Ramírez-Vélez 2021 (1)	24	.35	13.75617	26	-4.55	9.135727	0.42 [-0.14, 0.98]	10.07
Robinson Ramírez-Vélez 2021 (2)	24	-.27	13.626219	26	-4.55	9.135727	0.37 [-0.19, 0.93]	10.08
Robinson Ramírez-Vélez 2021 (3)	25	3.3	10.725462	26	-4.55	9.135727	0.78 [0.21, 1.35]	9.99
**Overall**							-0.05 [-0.43, 0.32]	

Heterogeneity: $\tau^2 = 0.28$, $I^2 = 75.81\%$, $H^2 = 4.13$

Test of $\theta_i = \theta_j$: Q(10) = 33.13, p = 0.00

Test of $\theta = 0$: z = -0.29, p = 0.77

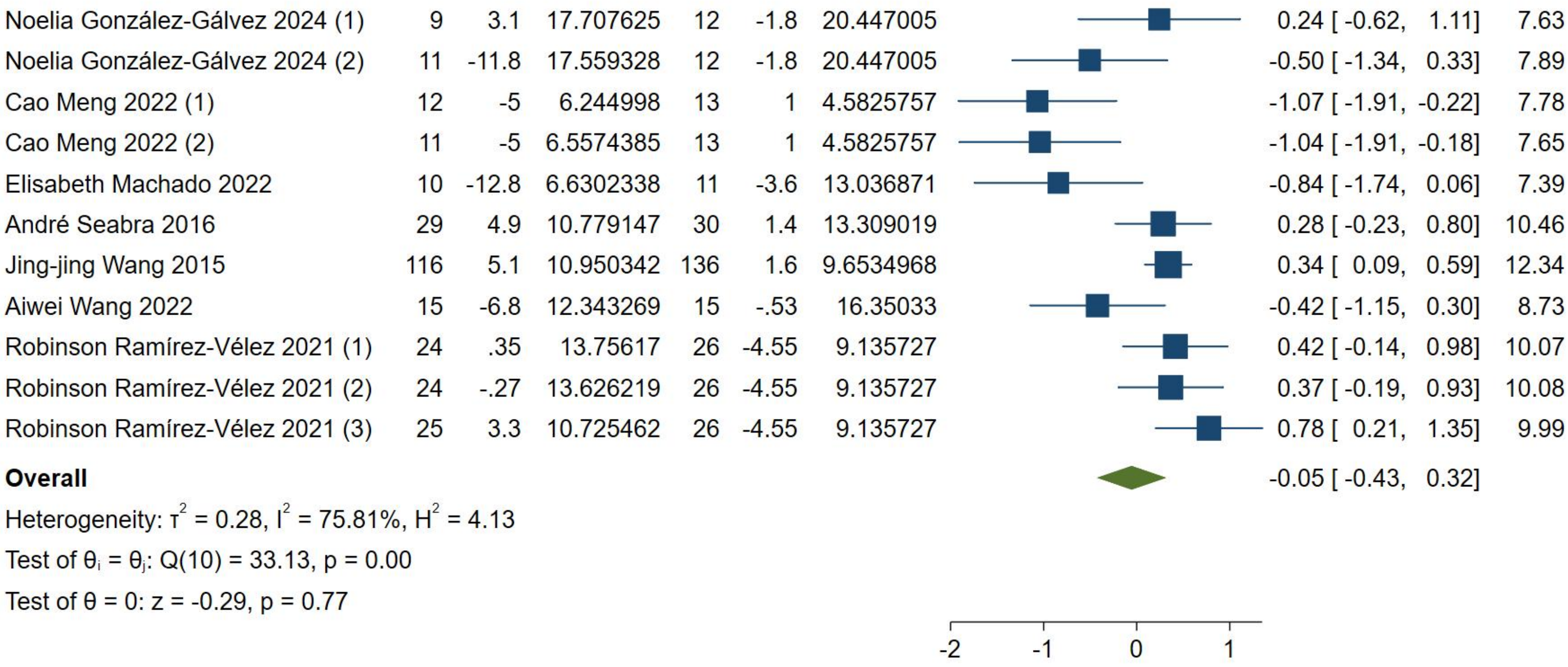

Random-effects REML model

Figure S7 Pooled effect size estimated for SBP

Study	Treatment N	Treatment Mean	Treatment SD	Control N	Control Mean	Control SD	Hedges'g with 95% CI	Weight (%)
Noelia González-Gálvez 2024 (1)	9	0	14.296503	12	-3.1	16.50818	0.19 [-0.68, 1.06]	5.57
Noelia González-Gálvez 2024 (2)	11	-13.7	14.082968	12	-3.1	16.50818	-0.66 [-1.51, 0.18]	5.80
Cao Meng 2022 (1)	12	-2	4.3588989	13	0	3.8587563	-0.47 [-1.27, 0.33]	6.35
Cao Meng 2022 (2)	11	-3	4	13	0	3.8587563	-0.74 [-1.57, 0.10]	5.91
Elisabeth Machado 2022	10	-6.3	7.3310027	11	1.4	13.143439	-0.69 [-1.57, 0.20]	5.37
André Seabra 2016	29	-4.8	7.8504777	30	1.8	8.981648	-0.77 [-1.30, -0.24]	11.14
Jing-jing Wang 2015	116	.5	8.2540899	136	2.6	7.0703607	-0.27 [-0.52, -0.03]	20.87
Aiwei Wang 2022	15	-2.46	17.344359	15	-.93	23.672795	-0.07 [-0.79, 0.64]	7.46
Robinson Ramírez-Vélez 2021 (1)	24	-4.07	8.2239989	26	-1.48	5.4962911	-0.37 [-0.93, 0.19]	10.42
Robinson Ramírez-Vélez 2021 (2)	24	-.05	8.2425632	26	-1.48	5.4962911	0.20 [-0.35, 0.76]	10.50
Robinson Ramírez-Vélez 2021 (3)	25	.32	6.473269	26	-1.48	5.4962911	0.30 [-0.26, 0.85]	10.60
**Overall**							-0.27 [-0.50, -0.04]	

Heterogeneity: $\tau^2 = 0.05$, $I^2 = 36.23\%$, $H^2 = 1.57$

Test of $\theta_i = \theta_j$: Q(10) = 14.85, p = 0.14

Test of $\theta = 0$: z = -2.33, p = 0.02

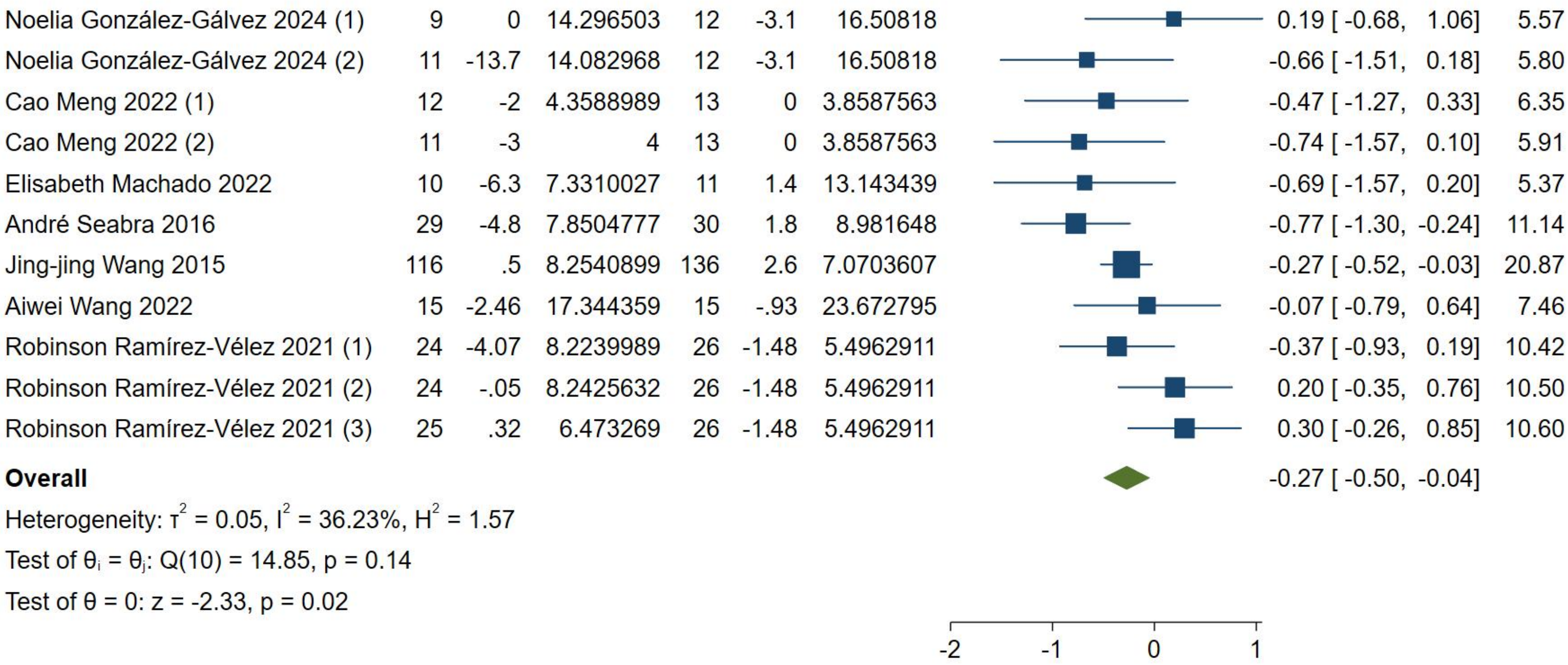

Random-effects REML model

Figure S8 Pooled effect size estimated for DBP

Study	Treatment N	Treatment Mean	Treatment SD	Control N	Control Mean	Control SD	Hedges'g with 95% CI	Weight (%)
Cao Meng 2022 (1)	12	7.732	10.228475	13	3.866	7.732	0.41 [-0.38, 1.21]	8.20
Cao Meng 2022 (2)	11	0	10.228475	13	3.866	7.732	-0.42 [-1.23, 0.40]	7.92
Elisabeth Machado 2022	10	.1	10.921996	11	-3.6	13.036871	0.29 [-0.57, 1.16]	7.27
André Seabra 2016	29	2.5	13.552491	30	2.3	8.3719771	0.02 [-0.49, 0.53]	14.23
Jing-jing Wang 2015	116	2.32	10.073768	136	-3.093	9.7572953	0.54 [0.29, 0.80]	23.38
Robinson Ramírez-Vélez 2021 (1)	24	-1.59	8.6138499	26	1.12	5.7067346	-0.37 [-0.93, 0.19]	12.88
Robinson Ramírez-Vélez 2021 (2)	24	2.37	8.5581569	26	1.12	5.7067346	0.17 [-0.39, 0.73]	12.98
Robinson Ramírez-Vélez 2021 (3)	25	2.17	6.7655159	26	1.12	5.7067346	0.17 [-0.38, 0.72]	13.14
**Overall**							0.15 [-0.12, 0.42]	

Heterogeneity: $\tau^2 = 0.06$, $I^2 = 46.27\%$, $H^2 = 1.86$

Test of $\theta_i = \theta_j$: Q(7) = 13.62, p = 0.06

Test of $\theta = 0$: z = 1.09, p = 0.28

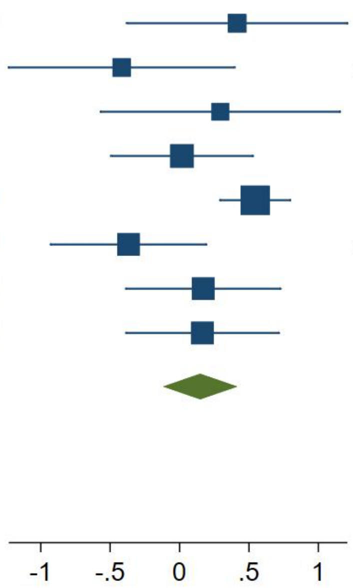

Random-effects REML model

Figure S9 Pooled effect size estimated for HDL-C

Study	Treatment N	Treatment Mean	Treatment SD	Control N	Control Mean	Control SD	Hedges'g with 95% CI	Weight (%)
Cao Meng 2022 (1)	12	-15.464	10.228475	13	3.866	19.33	-1.19 [-2.06, -0.33]	9.27
Cao Meng 2022 (2)	11	-11.598	13.392217	13	3.866	19.33	-0.88 [-1.73, -0.04]	9.44
Elisabeth Machado 2022	10	-15.2	26.031519	11	-7.7	24.184913	-0.29 [-1.15, 0.57]	9.29
André Seabra 2016	29	-6.4	30.814445	30	2	24.721044	-0.30 [-0.81, 0.22]	14.04
Jing-jing Wang 2015	116	8.118	17.409909	136	1.933	17.409909	0.35 [0.10, 0.60]	17.89
Robinson Ramírez-Vélez 2021 (1)	24	-7.59	22.481406	26	4.3	15.040526	-0.62 [-1.19, -0.05]	13.19
Robinson Ramírez-Vélez 2021 (2)	24	7.18	22.425713	26	4.3	15.040526	0.15 [-0.41, 0.71]	13.39
Robinson Ramírez-Vélez 2021 (3)	25	4.74	17.827061	26	4.3	15.040526	0.03 [-0.52, 0.58]	13.49
**Overall**							-0.26 [-0.62, 0.11]	

Heterogeneity: $\tau^2 = 0.18$, $I^2 = 70.07\%$, $H^2 = 3.34$

Test of $\theta_i = \theta_j$: Q(7) = 25.77, p = 0.00

Test of $\theta = 0$: z = -1.39, p = 0.16

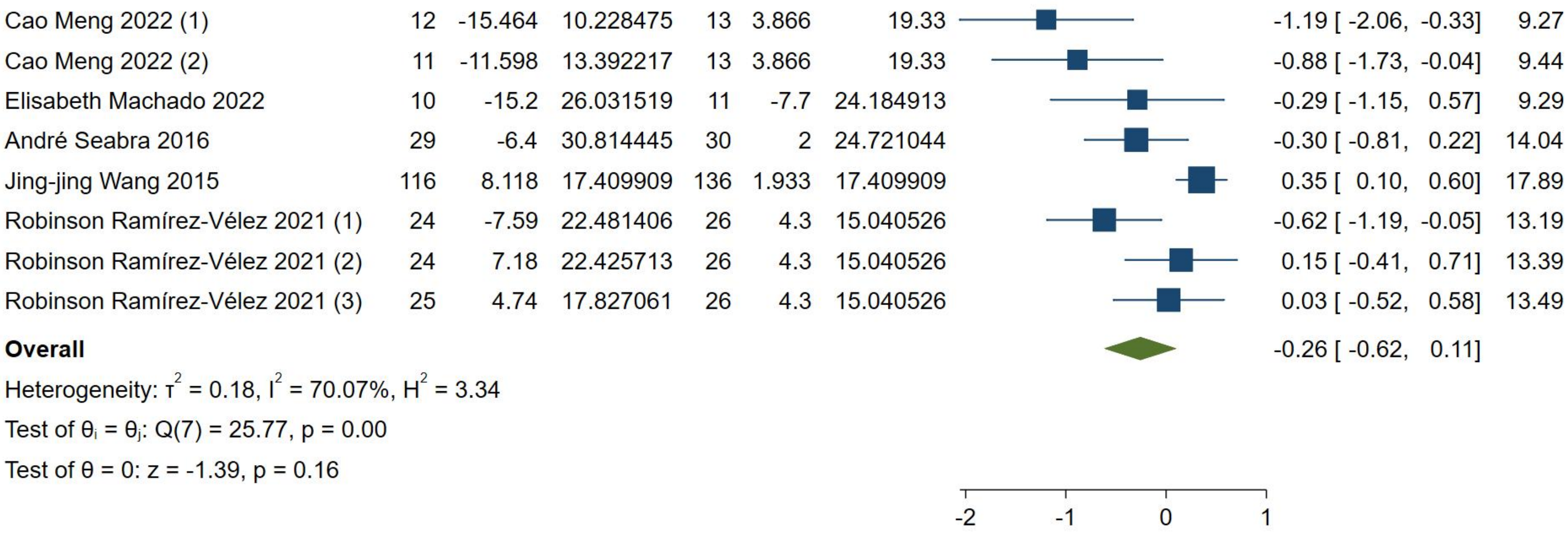

Random-effects REML model

Figure S10 Pooled effect size estimated for LDL-C

Study	Treatment N	Treatment Mean	Treatment SD	Control N	Control Mean	Control SD	Hedges'g with 95% CI	Weight (%)
Cao Meng 2022 (1)	12	-17.72	31.945184	13	17.72	49.330392	-0.82 [-1.64, 0.01]	7.22
Cao Meng 2022 (2)	11	-8.86	55.330682	13	17.72	49.330392	-0.49 [-1.31, 0.33]	7.30
Elisabeth Machado 2022	10	12.3	38.200393	11	50.9	120.93176	-0.40 [-1.27, 0.46]	6.63
André Seabra 2016	29	-23	47.83482	30	12.2	38.852413	-0.80 [-1.33, -0.27]	13.61
Jing-jing Wang 2015	116	-1.772	61.441401	136	5.316	47.02482	-0.13 [-0.38, 0.12]	27.01
Robinson Ramírez-Vélez 2021 (1)	24	-.62	64.58531	26	10.41	43.128554	-0.20 [-0.76, 0.36]	12.84
Robinson Ramírez-Vélez 2021 (2)	24	10.08	64.566745	26	10.41	43.128554	-0.01 [-0.56, 0.55]	12.88
Robinson Ramírez-Vélez 2021 (3)	25	-23.27	51.055535	26	10.41	43.128554	-0.70 [-1.27, -0.14]	12.52
**Overall**							-0.38 [-0.63, -0.13]	

Heterogeneity: $\tau^2 = 0.04$, $I^2 = 36.23\%$, $H^2 = 1.57$

Test of $\theta_i = \theta_j$: Q(7) = 10.10, p = 0.18

Test of $\theta = 0$: z = -3.02, p = 0.00

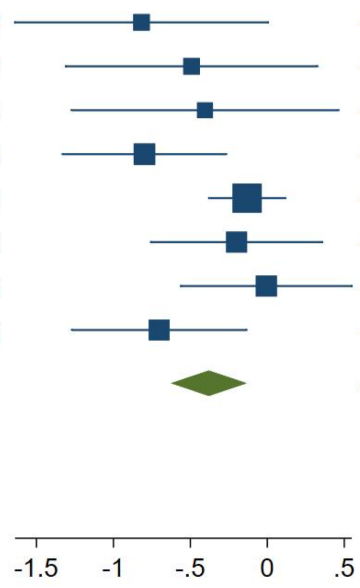

Random-effects REML model

Figure S11 Pooled effect size estimated for TG

Study	Treatment N	Treatment Mean	Treatment SD	Control N	Control Mean	Control SD	Hedges'g with 95% CI	Weight (%)
Cao Meng 2022 (1)	12	0	21.524977	13	7.732	21.524977	-0.35 [-1.14, 0.44]	11.89
Cao Meng 2022 (2)	11	-11.598	27.062	13	7.732	21.524977	-0.77 [-1.61, 0.07]	11.39
André Seabra 2016	29	-8.4	29.173275	30	6.7	23.556103	-0.56 [-1.08, -0.04]	15.07
Jing-jing Wang 2015	116	-3.479	23.295515	136	-22.81	24.053061	0.81 [0.56, 1.07]	17.81
Robinson Ramírez-Vélez 2021 (1)	24	-13.15	25.748728	26	10.41	43.128554	-0.65 [-1.22, -0.08]	14.48
Robinson Ramírez-Vélez 2021 (2)	24	4.41	25.618778	26	10.41	43.128554	-0.16 [-0.72, 0.39]	14.65
Robinson Ramírez-Vélez 2021 (3)	25	1.79	25.897243	26	10.41	43.128554	-0.24 [-0.79, 0.31]	14.71
**Overall**							-0.22 [-0.67, 0.23]	

Heterogeneity: $\tau^2 = 0.28$, $I^2 = 79.94\%$, $H^2 = 4.99$

Test of $\theta_i = \theta_j$: Q(6) = 49.34, p = 0.00

Test of $\theta = 0$: z = -0.97, p = 0.33

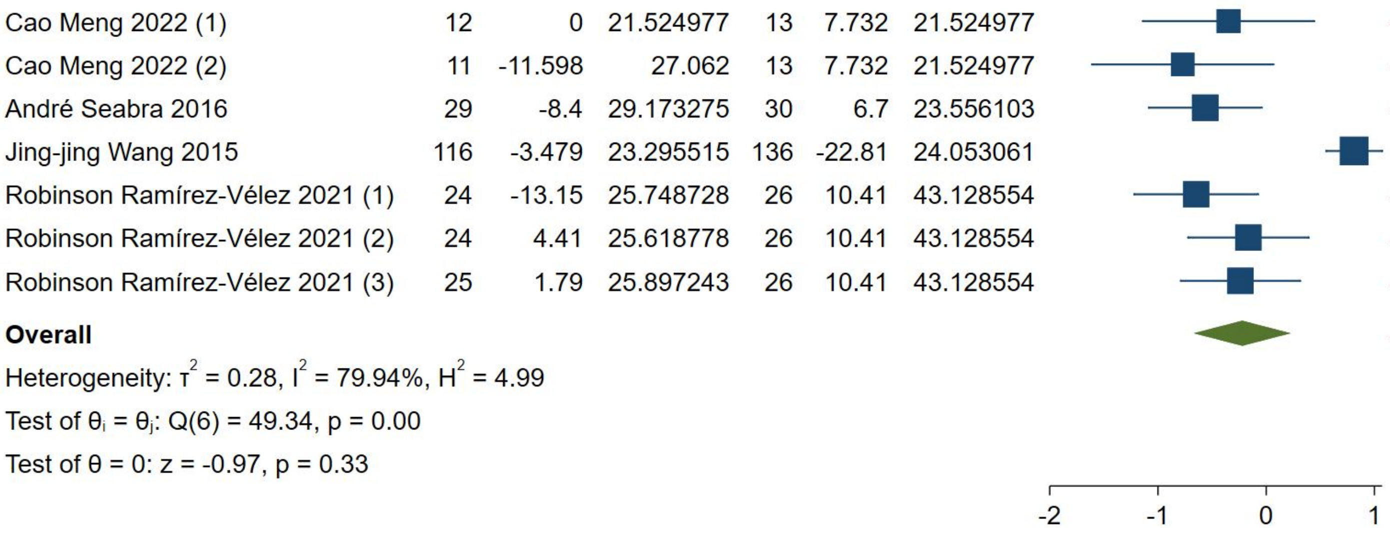

Random-effects REML model

Figure S12 Pooled effect size estimated for TC

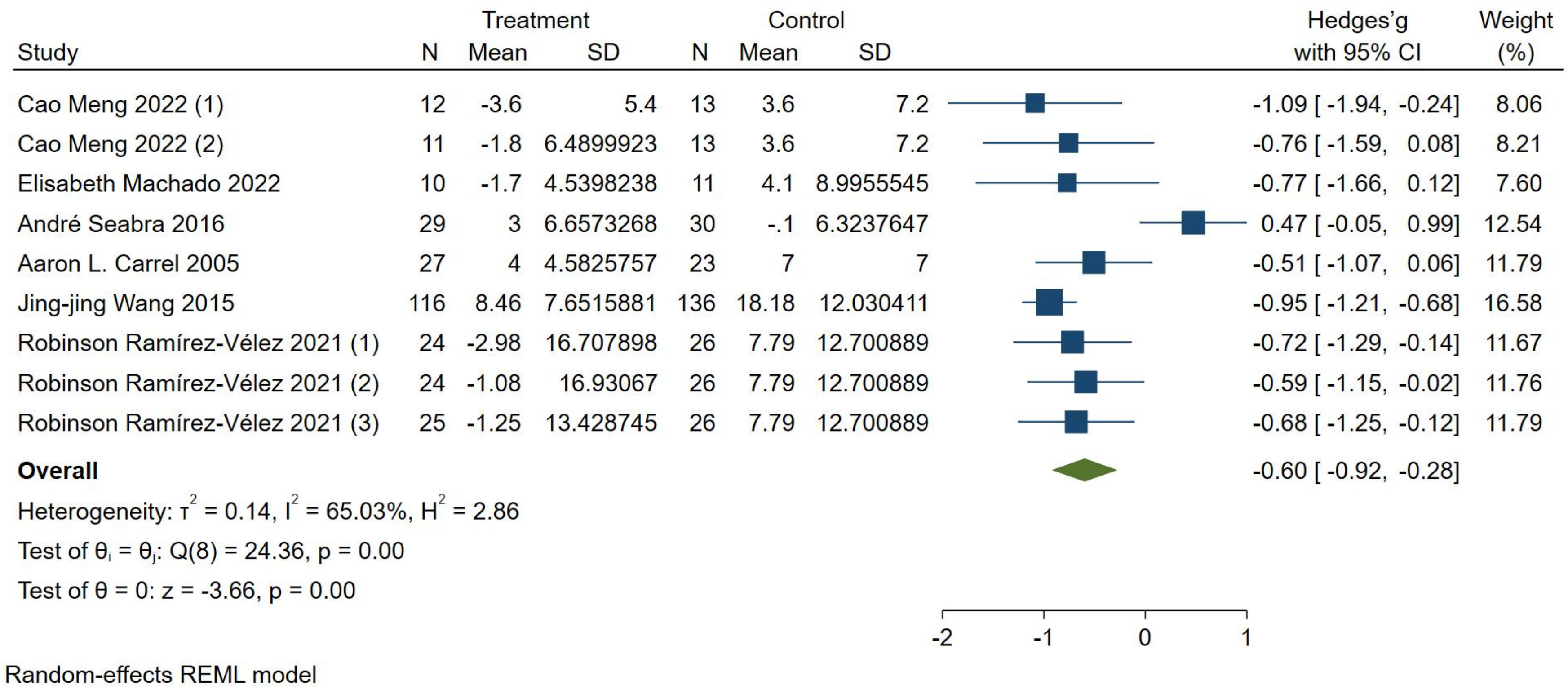

Figure S13 Pooled effect size estimated for FBG

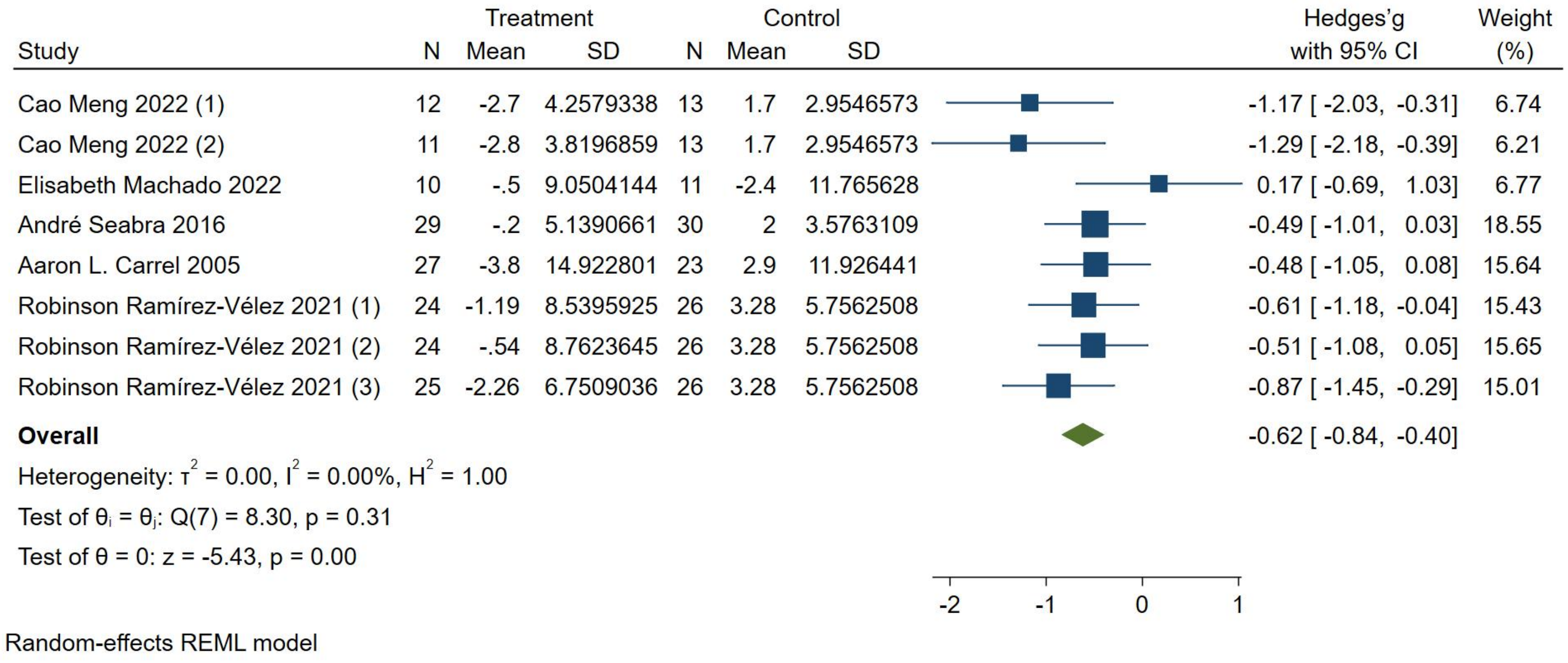

Figure S14 Pooled effect size estimated for BI

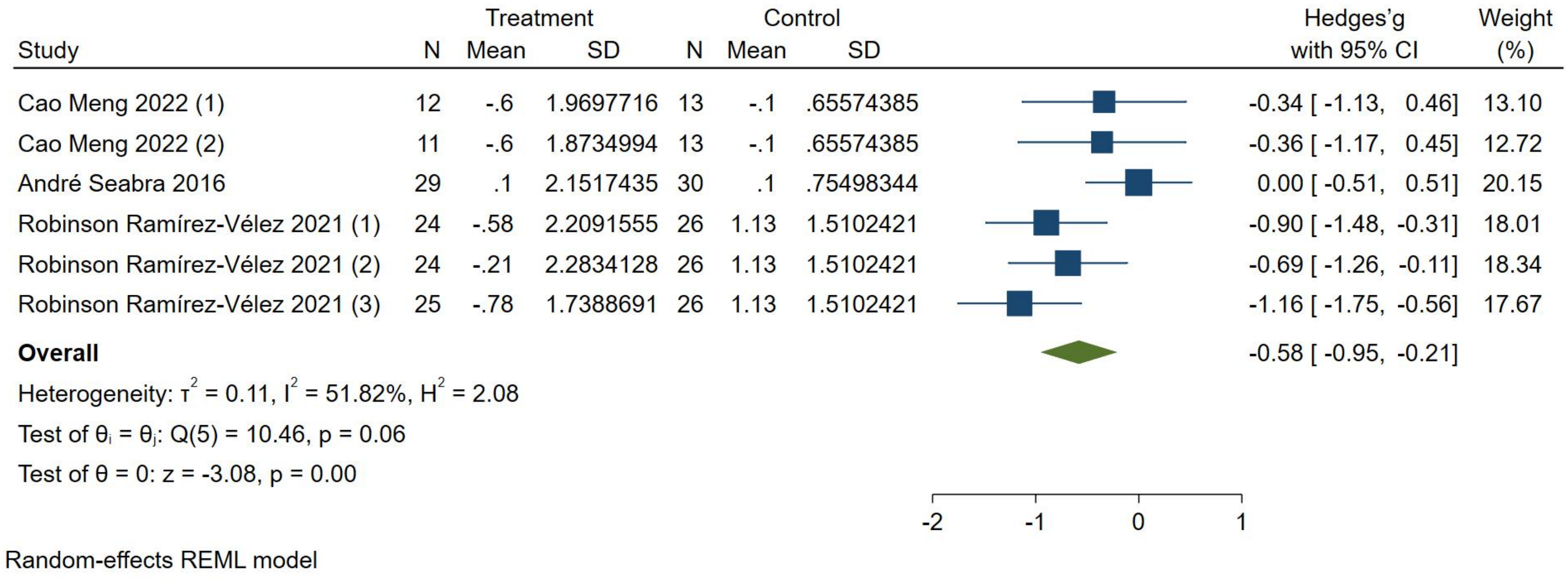

Figure S15 Pooled effect size estimated for HOMA-IR

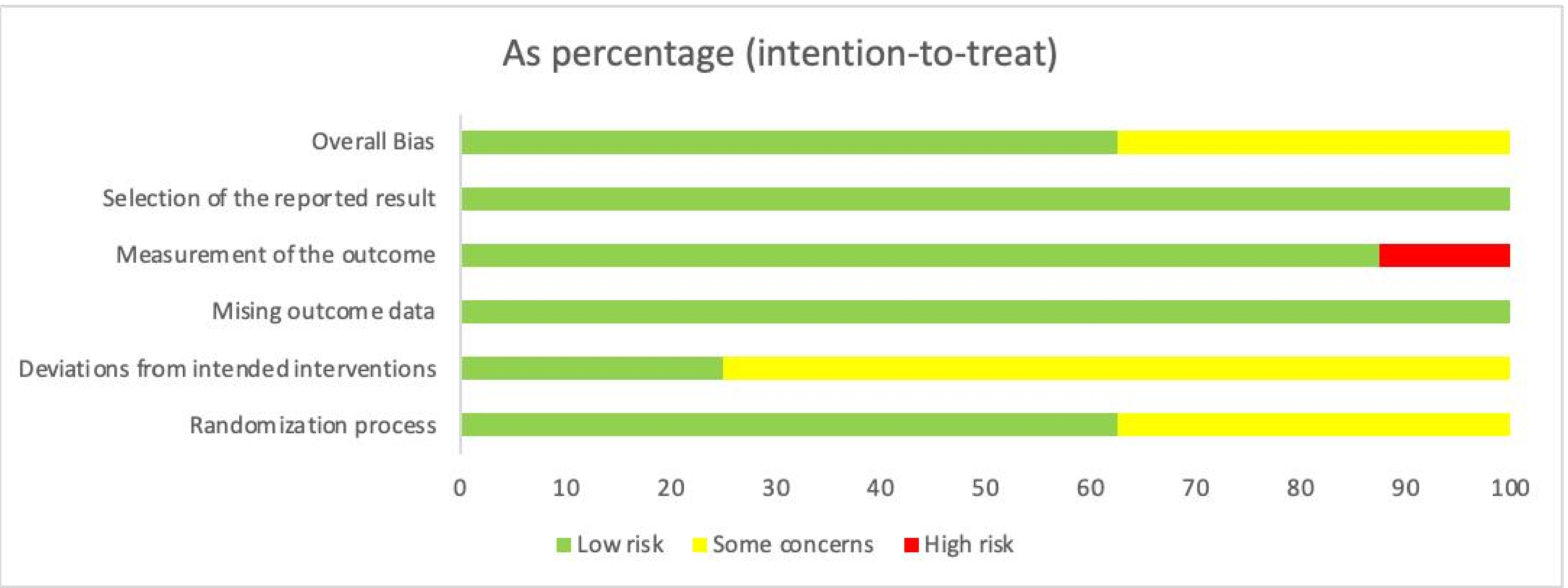

Figure S16 Risk of Bias in RCTs Assessment Using RoB 2 Tool

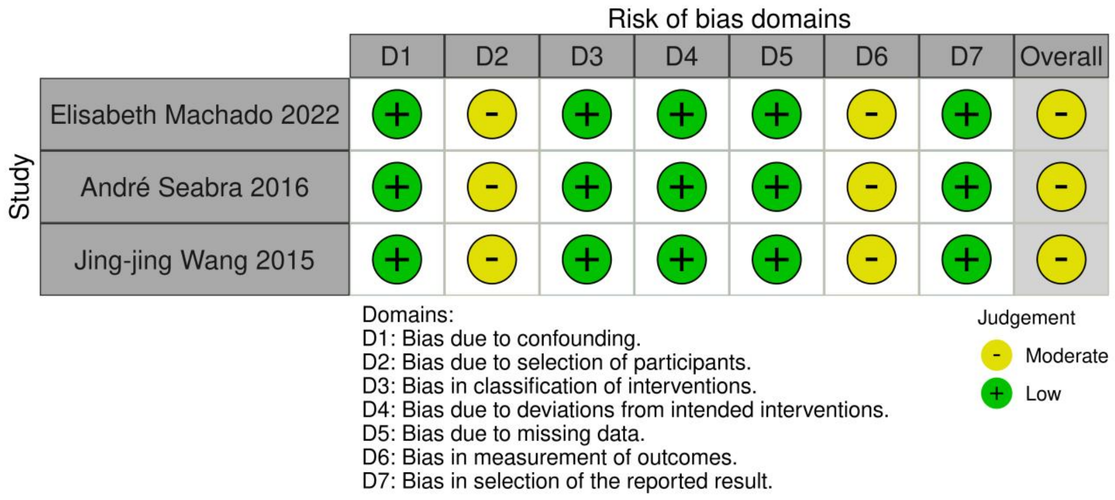

Figure S17 Risk of Bias in non-RCTs Assessment Using ROBINS-I Tool
