# Supplementary material for: Evaluating the Role of School-Based Physical Activity in Mitigating Cardiometabolic Risk Factors in Children and Adolescents with Overweight or Obesity: A Systematic Review and Meta-Analysis
Source: Children (Basel). 2025 Mar 29;12(4):439. doi: 10.3390/children12040439 (PMC12025731; doi:10.3390/children12040439)
Supplement: Supplementary file 1 [file children-12-00439-s001.zip › Supplementary Tables.pdf]

Supplementary Tables

Table S1. RoB2 general scores for RCTs

Study ID	Randomization process	Deviations from intended interventions	Mising outcome data	Measurement of the outcome	Selection of the reported result	Overall Bias
Noelia González-Gálvez (2024)	Low	Low	Low	Low	Low	Low
Cao Meng（2022）	Low	Some concerns	Low	Low	Some concerns	Some concerns
Sumathy Ponnambalam (2022)	Some concerns	Some concerns	Low	High	Low	Some concerns
Danielle Lambrick (2016)	Some concerns	Some concerns	Low	Low	Low	Some concerns
Aaron L. Carrel (2005)	Some concerns	Some concerns	Low	Low	Low	Some concerns
Aiwei Wang (2022)	Low	Low	Low	Low	Low	Low
Siyue Yu (2022)	Low	Some concerns	Low	Low	Low	Low
Robinson Ramírez-Vélez (2021)	Low	Some concerns	Low	Low	Low	Low

Table S2. ROBINS-I general scores for the non-RCTs

Study ID	Confounding	Selection of Participants into the Study	Classification of Interventions	Deviations from Intended Interventions	Missing Data	Measurement of Outcomes	Selection of the Reported Results	Overall Bias
Elisabeth Machado 2022	Low	Moderate	Low	Low	Low	Moderate	Low	moderate
André Seabra 2016	Low	Moderate	Low	Low	Low	Moderate	Low	moderate
Jing-jing Wang 2015	Low	Moderate	Low	Low	Low	Moderate	Low	moderate
